# Supplementary material for: ERBB3-dependent AKT and ERK pathways are essential for atrioventricular cushion development in mouse embryos
Source: PLoS One. 2021 Oct 29;16(10):e0259426. doi: 10.1371/journal.pone.0259426 (PMC8555822; doi:10.1371/journal.pone.0259426)

***Supplementary data***

**Supplementary figures**

**
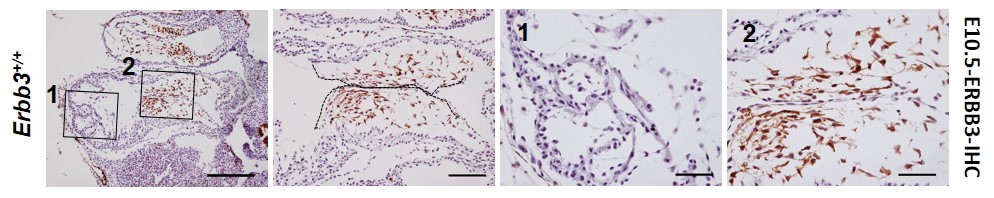
** **Figure S1. Histological ERBB3 expression in the heart of the wild-type E10.5.** Section 1 represents the trabeculated myocardium, and section 2 represents the AV cushion.

**
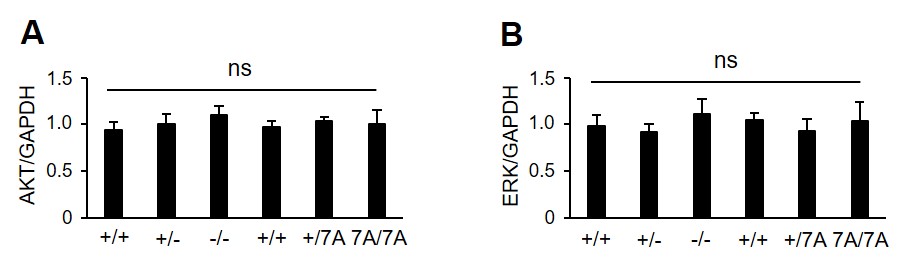
**

**Fig. S2. Quantification and comparison of AKT and ERK expression.** ns, non-significant.

Fig. S2A

| AKT/GAPDH |  |  |  |  |  |
| --- | --- | --- | --- | --- | --- |
| +/+ | +/- | -/- | +/+ | +/7A | 7A/7A |
| 1.032 | 0.969 | 0.994 | 1.021 | 0.973 | 1.004 |
| 0.911 | 0.907 | 1.195 | 0.882 | 1.079 | 0.914 |
| 0.859 | 1.120 | 1.109 | 0.999 | 1.041 | 0.889 |
|  |  |  | 0.996 | 1.046 | 1.208 |
| Fig. S2B  ERK/GAPDH |  |  |  |  |  |
| +/+ | +/- | -/- | +/+ | +/7A | 7A/7A |
| 0.916 | 0.977 | 1.123 | 1.030 | 0.981 | 0.987 |
| 0.911 | 0.824 | 0.953 | 1.110 | 1.073 | 0.960 |
| 1.112 | 0.948 | 1.263 | 1.093 | 0.871 | 0.846 |
|  |  |  | 0.955 | 0.780 | 1.328 |

**
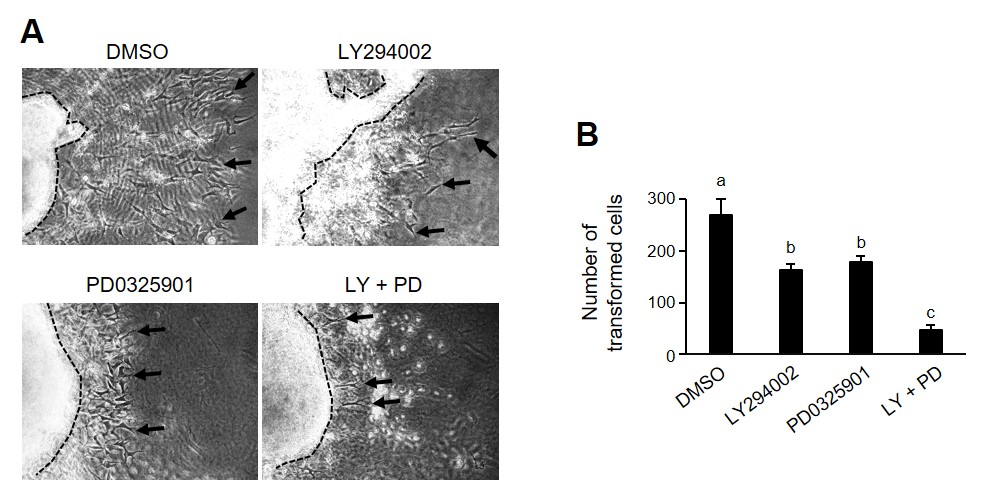
**

**Fig. S3.** **Collagen gel analysis using E9.5 embryo heart.** (A) Collagen gel analysis was performed with AVC-explants from wild-type embryos at E9.5 for 48 h in the presence of DMSO (vehicle alone), LY294002 (3 μM), PD0325901 (10 nM), and LY + PD (3 μM of LY294002 and 10 nM of PD0325901), respectively. (B) The number of transformed cells was counted under the inverted microscope (n = 6-9). Different letters on the bars represent the statistically significant difference, *p* < 0.05.

Fig. S3B

| **DMSO** | **LY294002-3 uM** | **PD0325901-10 nM** | **LY294002-3 uM + PD0325901-10 nM** |
| --- | --- | --- | --- |
| 293 | 79 | 169 | 70 |
| 297 | 118 | 250 | 54 |
| 214 | 151 | 175 | 17 |
| 257 | 170 | 124 |  |
| 248 |  | 137 |  |
| 232 |  | 210 |  |
| 169 |  | 35 |  |
| 494 |  |  |  |


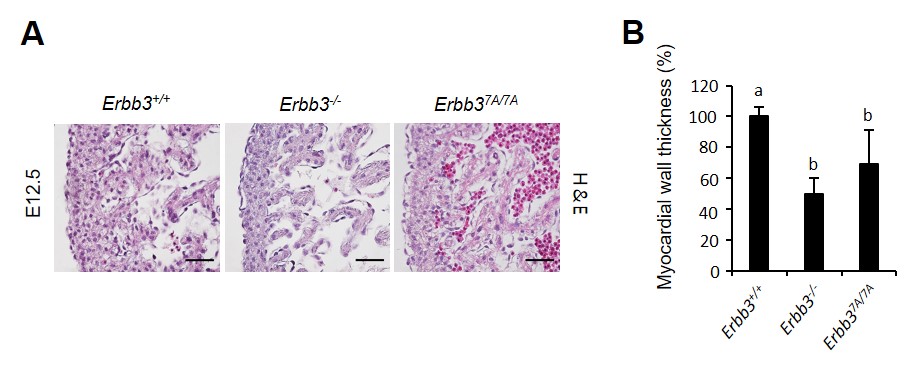
 **Figure. S4. Myocardial wall thickness of E12.5 embryos.** (A) At E12.5, the myocardial wall of the heart was examined by hematoxylin and eosin (H&E) staining. Scale bar = 50 µm. (B) Quantification of myocardial wall thickness in each samples of *Erbb3^+/+^*, *Erbb3^-/-^*, and *Erbb3^7A/7A^*.

Fig. S4B

| *Erbb3^+/+^* | *Erbb3^-/-^* | *Erbb3^7A/7A^* |
| --- | --- | --- |
| 104.514 | 65.150 | 64.676 |
| 105.070 | 46.851 | 94.867 |
| 92.208 | 44.132 | 41.898 |
| 98.209 | 43.258 | 75.576 |

**Figure 1.**

Fig. 1B


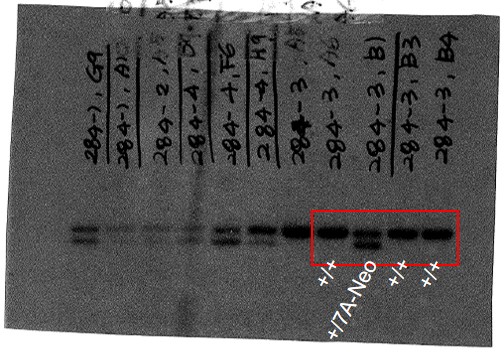


Fig. 1E


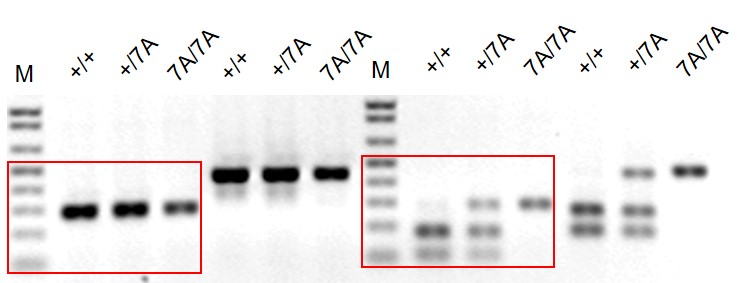


Fig. 1F

| *Erbb3^+/+^* |  |  |  |
| --- | --- | --- | --- |
| ERBB1 | ERBB2 | ERBB3 | ERBB4 |
| 0.964 | 0.548 | 1.0290 | 0.943 |
| 1.036 | 1.339 | 0.9710 | 1.057 |
| 1.251 | 1.424 | 1.2837 | 1.357 |
| 0.749 | 0.576 | 0.7163 | 0.643 |
| *Erbb3^+/7A^* | |  |  |
| ERBB1 | ERBB2 | ERBB3 | ERBB4 |
| 0.751 | 1.352 | 1.130 | 0.896 |
| 1.330 | 0.981 | 1.209 | 0.927 |
| 0.989 | 0.961 | 0.888 | 0.877 |
| 0.993 | 1.019 | 0.951 | 0.829 |
| 1.011 | 1.252 | 1.112 | 1.123 |
| 1.186 | 1.436 | 0.882 | 0.995 |
| 1.148 |  | 1.079 |  |
| *Erbb3^7A/7A^* | |  |  |
| ERBB1 | ERBB2 | ERBB3 | ERBB4 |
| 1.024 | 0.995 | 1.093 | 0.907 |
| 1.049 | 0.945 | 1.195 | 0.989 |
| 1.101 | 1.463 | 1.360 | 1.117 |
| 1.015 |  |  |  |
| 1.150 |  |  |  |

Fig. 1G

Western blot data


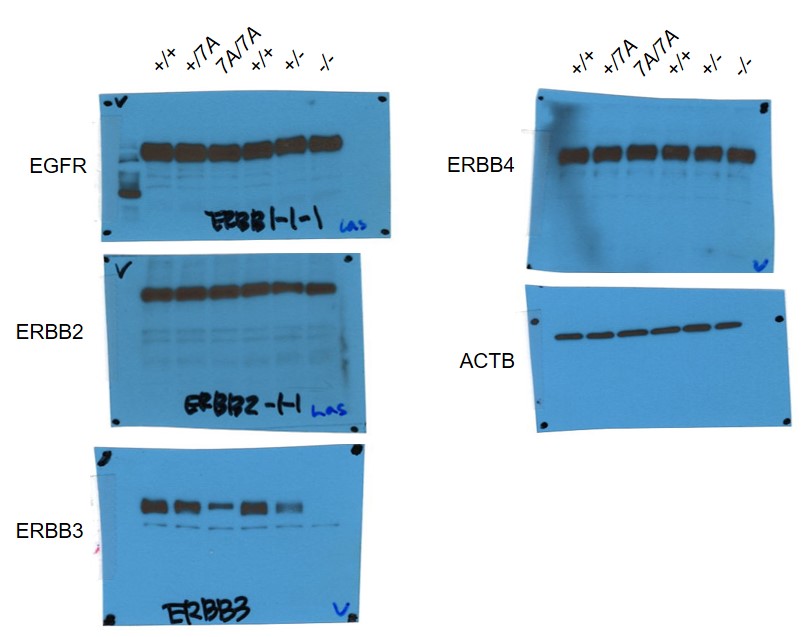


| ERBB1/ACTB |  |  |  |  |
| --- | --- | --- | --- | --- |
| +/+ | 1.000 | 1.000 | 1.000 | 1.000 |
| +/7A | 0.906 | 1.074 | 1.058 | 0.906 |
| 7A/7A | 0.940 | 0.907 | 0.984 | 0.819 |
| +/+ | 0.940 | 0.833 | 1.096 | 0.946 |
| +/- | 0.868 | 1.006 | 0.993 | 0.854 |
| -/- | 1.149 | 0.988 | 0.860 | 0.856 |
| ERBB2/ACTB |  |  |  |  |
| +/+ | 1.000 | 1.000 | 1.000 | 1.000 |
| +/7A | 0.937 | 0.995 | 0.938 | 1.163 |
| 7A/7A | 0.974 | 0.770 | 0.932 | 1.236 |
| +/+ | 0.944 | 0.916 | 1.038 | 1.392 |
| +/- | 0.745 | 0.813 | 1.089 | 1.181 |
| -/- | 0.995 | 0.644 | 0.904 | 1.153 |
| ERBB3/ACTB |  |  |  |  |
| +/+ | 1.000 | 1.000 | 1.000 | 1.000 |
| +/7A | 0.777 | 0.698 | 0.779 | 0.842 |
| 7A/7A | 0.421 | 0.359 | 0.317 | 0.235 |
| +/+ | 0.882 | 0.948 | 1.042 | 1.017 |
| +/- | 0.318 | 0.447 | 0.383 | 0.533 |
| -/- | 0 | 0 | 0 | 0 |
| ERBB4/ACTB |  |  |  |  |
| +/+ | 1.000 | 1.000 | 1.000 | 1.000 |
| +/7A | 1.002 | 0.908 | 0.867 | 0.867 |
| 7A/7A | 1.194 | 0.921 | 0.849 | 0.790 |
| +/+ | 0.976 | 0.977 | 0.968 | 0.899 |
| +/- | 0.750 | 1.064 | 0.883 | 0.805 |
| -/- | 1.223 | 1.048 | 0.832 | 0.820 |

Fig. 1H

Western blot data

**
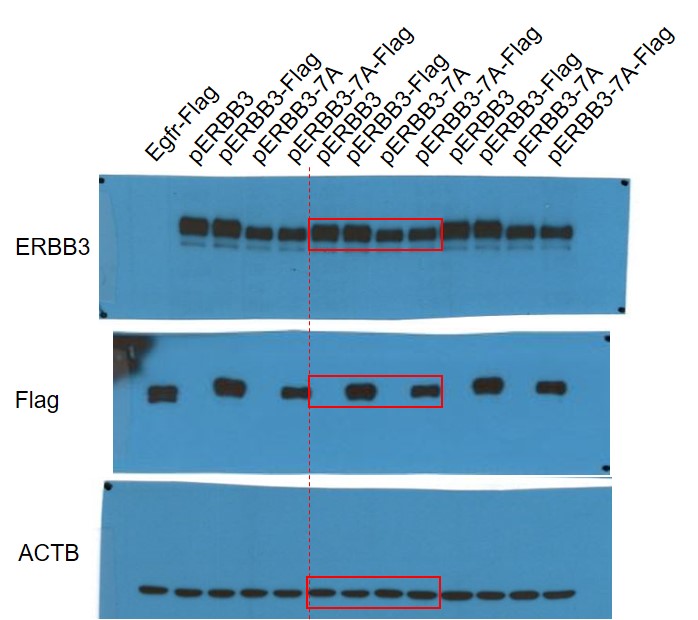
**

Density of Western blot

| pERBB3-Flag (n=3) | pERBB3^7A^-Flag (n=3) |
| --- | --- |
| 1.000 | 0.660 |
| 0.840 | 0.560 |
| 0.880 | 0.470 |

**Figure 3.**

Fig. 3B

| **E10.5** |  |  |  |  | **E11.5** |  |  |  |
| --- | --- | --- | --- | --- | --- | --- | --- | --- |
| **+/+** | **+/-** | **-/-** | **7A/7A** |  | **+/+** | **+/-** | **-/-** | **7A/7A** |
| 147 | 155 | 97 | 107 |  | 287 | 152 | 175 | 137 |
| 117 | 158 | 84 | 119 |  | 174 | 274 | 144 | 202 |
| 161 | 149 | 71 | 83 |  | 215 | 210 | 99 | 230 |
| 145 | 137 | 87 | 80 |  | 258 | 196 | 217 | 69 |
| 151 |  | 49 | 106 |  | 230 | 211 | 187 | 135 |
| 153 |  | 114 |  |  | 196 | 149 | 115 | 180 |
| 151 |  | 108 |  |  |  | 218 |  |  |
|  |  |  |  |  |  | 168 |  |  |

Fig. 3C

| **E10.5** |  |  |  |  | **E11.5** |  |  |  |
| --- | --- | --- | --- | --- | --- | --- | --- | --- |
| **+/+** | **+/-** | **-/-** | **7A/7A** |  | **+/+** | **+/-** | **-/-** | **7A/7A** |
| 4 | 6 | 6 | 4 |  | 6 | 5 | 6 | 5 |
| 5 | 4 | 4 | 4 |  | 5 | 5 | 5 | 5 |
| 4 | 4 | 5 | 5 |  | 6 | 5 | 6 | 5 |
| 5 | 4 | 5 | 4 |  | 7 | 6 | 5 | 7 |
| 4 | 5 | 4 | 5 |  | 7 | 6 |  |  |
| 4 | 4 | 4 | 5 |  |  |  |  |  |
|  | 5 |  | 5 |  |  |  |  |  |

Fig. 3D

| +/+ | +/- | -/- | 7A/7A |
| --- | --- | --- | --- |
| 191 | 221 | 15 | 224 |
| 218 | 233 | 47 | 91 |
| 293 | 261 | 218 | 151 |
| 207 | 114 | 45 | 105 |
| 421 | 128 | 138 | 31 |
| 295 | 298 | 84 | 46 |
| 408 | 240 | 101 | 204 |
| 130 | 218 |  |  |
| 100 |  |  |  |
| 411 |  |  |  |
| 421 |  |  |  |
| 262 |  |  |  |

**Figure 4.**

Fig. 4B

| **E10.5** |  |  |  |  | **E11.5** |  |  |  |
| --- | --- | --- | --- | --- | --- | --- | --- | --- |
| **+/+** | **+/-** | **-/-** | **7A/7A** |  | **+/+** | **+/-** | **-/-** | **7A/7A** |
| 33 | 30 | 33 | 38 |  | 36 | 38 | 31 | 43 |
| 52 | 32.4 | 31.4 | 26 |  | 34 | 38 | 41 | 42 |
| 39 | 31.5 | 29.7 | 22 |  | 32 | 41 | 36 | 28 |
| 55.3 | 40 | 24.4 | 24 |  | 41 | 41 | 32 | 36 |
| 35 | 49 | 26.3 | 37.8 |  | 37 | 42 | 40 | 35 |
| 43.2 |  | 35.1 | 36 |  | 40 | 45 | 39 |  |
| 43 |  | 27 | 35 |  | 40 |  |  |  |

Fig. 4C

| **E10.5** |  |  |  |  | **E11.5** |  |  |  |
| --- | --- | --- | --- | --- | --- | --- | --- | --- |
| **+/+** | **+/-** | **-/-** | **7A/7A** |  | **+/+** | **+/-** | **-/-** | **7A/7A** |
| 26 | 31 | 34 | 48 |  | 47 | 48 | 12 | 19 |
| 33 | 37 | 30 | 14 |  | 33 | 35 | 20 | 27 |
| 29 | 12 | 22 | 33 |  | 24 | 21 | 22 | 11 |
| 34 | 34 | 14 | 32 |  | 35 | 34 | 19 | 17 |
| 27 | 23 | 38 | 22 |  | 35 | 34 | 16 |  |
| 15 | 25 | 33 | 40 |  | 33 | 51 |  |  |
| 62 |  |  | 27 |  |  | 17 |  |  |
|  |  |  | 30 |  |  |  |  |  |

**Figure 5.**

Fig. 5A

Western blot data


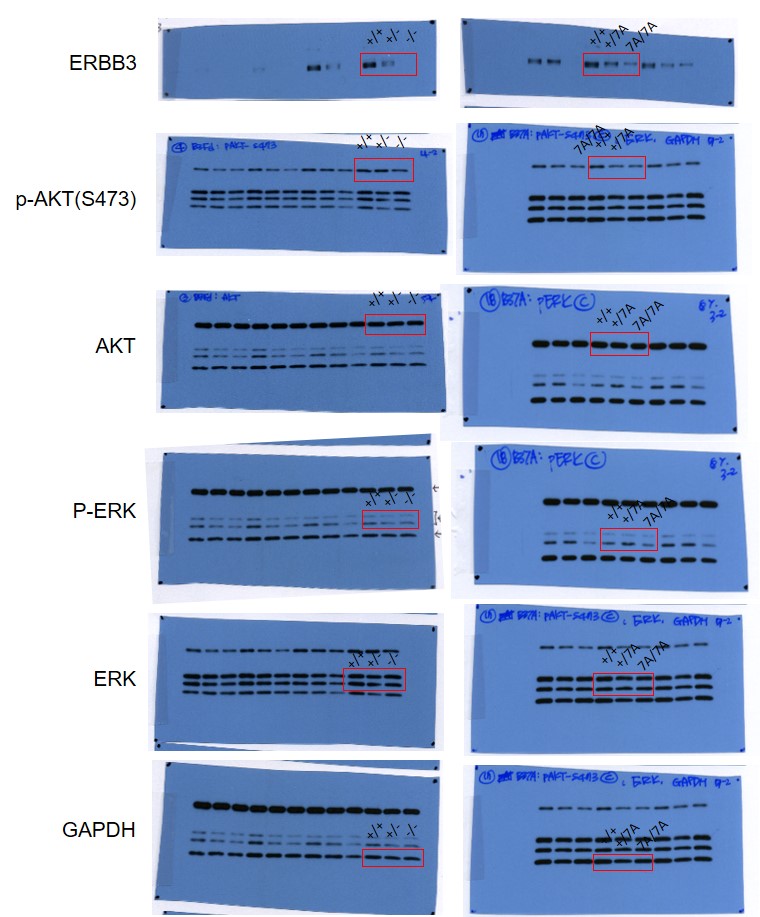


| pAKT-S473/AKT |  |  |  |  |  |  |
| --- | --- | --- | --- | --- | --- | --- |
| +/+ | 1.000 | 1.000 | 1.000 | 1.000 | 1.000 |  |
| +/- | 1.277 | 0.884 | 1.348 | 0.958 | 1.117 |  |
| -/- | 0.776 | 0.533 | 0.918 | 0.743 |  |  |
| +/+ | 1.000 | 1.000 | 1.000 | 1.000 | 1.000 | 1.000 |
| +/7A | 1.043 | 0.810 | 0.819 | 1.179 |  |  |
| 7A/7A | 0.450 | 0.629 | 0.533 | 0.866 | 0.347 | 0.415 |
| pERK/ERK |  |  |  |  |  |  |
| +/+ | 1.000 | 1.000 | 1.000 | 1.000 | 1.000 | 1.000 |
| +/- | 0.880 | 0.824 | 0.950 | 1.285 | 1.258 |  |
| -/- | 0.345 | 0.925 | 0.327 | 0.574 |  |  |
| +/+ | 1.000 | 1.000 | 1.000 | 1.000 | 1.000 | 1.000 |
| +/7A | 0.607 | 1.143 | 1.164 | 1.317 |  |  |
| 7A/7A | 0.376 | 0.348 | 0.896 | 0.383 | 0.510 | 1.015 |

Fig. 5B

| E10.5 |  |  |  |  |  |  |  |  |
| --- | --- | --- | --- | --- | --- | --- | --- | --- |
| Endocardial | |  |  |  | Mesenchymal | |  |  |
| **+/+** | **+/-** | **-/-** | **7A/7A** |  | **+/+** | **+/-** | **-/-** | **7A/7A** |
| 1.00 | 1.00 | 0.97 | 0.51 |  | 1.00 | 1.00 | 0.85 | 0.83 |
| 1.00 | 1.25 | 0.47 | 1.00 |  | 1.00 | 1.27 | 0.74 | 0.57 |
| 1.00 | 0.87 | 0.80 | 0.56 |  | 1.00 | 1.31 | 0.79 | 0.39 |
| 1.00 | 1.04 | 0.12 | 0.80 |  | 1.00 | 0.77 | 0.53 | 0.66 |
| 1.00 | 1.19 | 0.48 | 0.90 |  | 1.00 | 0.69 | 0.71 |  |
| 1.00 | 0.64 | 0.52 |  |  | 1.34 | 0.99 | 0.46 |  |
| 0.86 | 0.61 | 0.73 |  |  | 0.66 | 1.01 | 0.59 |  |
| 1.14 | 0.83 | 0.71 |  |  | 1.06 |  |  |  |
|  |  | 0.35 |  |  | 0.94 |  |  |  |
|  |  |  |  |  |  |  |  |  |
| E11.5 |  |  |  |  |  |  |  |  |
| Endocardial | |  |  |  | Mesenchymal | |  |  |
| **+/+** | **+/-** | **-/-** | **7A/7A** |  | **+/+** | **+/-** | **-/-** | **7A/7A** |
| 1.16 | 1.10 | 0.60 | 0.58 |  | 1.26 | 0.92 | 0.42 | 0.56 |
| 0.84 | 0.98 | 0.76 | 0.92 |  | 0.74 | 0.89 | 0.60 | 0.65 |
| 1.00 | 0.68 | 0.81 | 0.69 |  | 1.00 | 0.95 | 0.76 | 0.56 |
| 1.00 | 0.72 | 0.69 | 0.59 |  | 1.00 | 0.62 | 0.77 | 0.60 |
| 1.00 |  | 0.75 | 0.79 |  | 1.00 | 0.79 | 0.72 | 0.29 |
| 1.00 |  |  | 0.43 |  | 1.00 |  | 0.73 |  |
| 1.00 |  |  |  |  | 1.00 |  | 0.64 |  |
| 0.97 |  |  |  |  | 0.97 |  |  |  |
| 1.03 |  |  |  |  | 1.03 |  |  |  |

Fig. 5C

| E10.5 |  |  |  |  |  |  |  |  |
| --- | --- | --- | --- | --- | --- | --- | --- | --- |
| Endocardial | |  |  |  | Mesenchymal | |  |  |
| **+/+** | **+/-** | **-/-** | **7A/7A** |  | **+/+** | **+/-** | **-/-** | **7A/7A** |
| 1.19 | 1.25 | 0.67 | 0.72 |  | 0.94 | 1.17 | 0.73 | 0.80 |
| 0.81 | 1.15 | 0.88 | 0.51 |  | 1.06 | 0.95 | 0.72 | 0.52 |
| 1.00 | 0.58 | 0.53 | 0.46 |  | 1.00 | 0.92 | 0.79 | 1.07 |
| 1.00 | 1.00 | 0.31 |  |  | 1.00 | 1.00 | 0.95 | 0.31 |
| 1.00 | 1.00 | 0.66 |  |  | 1.00 | 1.00 | 0.94 |  |
|  | 1.36 |  |  |  | 1.00 | 1.18 | 0.75 |  |
| E11.5 |  |  |  |  |  |  |  |  |
| Endocardial | |  |  |  | Mesenchymal | |  |  |
| **+/+** | **+/-** | **-/-** | **7A/7A** |  | **+/+** | **+/-** | **-/-** | **7A/7A** |
| 1.14 | 1.07 | 0.58 | 0.76 |  | 1.06 | 1.06 | 0.61 | 0.95 |
| 0.86 | 0.52 | 0.51 | 0.81 |  | 0.94 | 0.75 | 0.50 | 0.77 |
| 1.00 | 1.26 | 0.62 | 0.63 |  | 1.00 | 0.99 | 0.49 | 0.59 |
| 1.00 | 0.74 | 0.92 | 0.21 |  | 1.00 | 1.01 | 0.46 | 0.76 |
|  | 0.48 |  | 0.31 |  | 1.00 |  |  | 0.53 |
|  |  |  |  |  |  |  |  | 0.35 |

**Figure 6.**

Fig. 6A


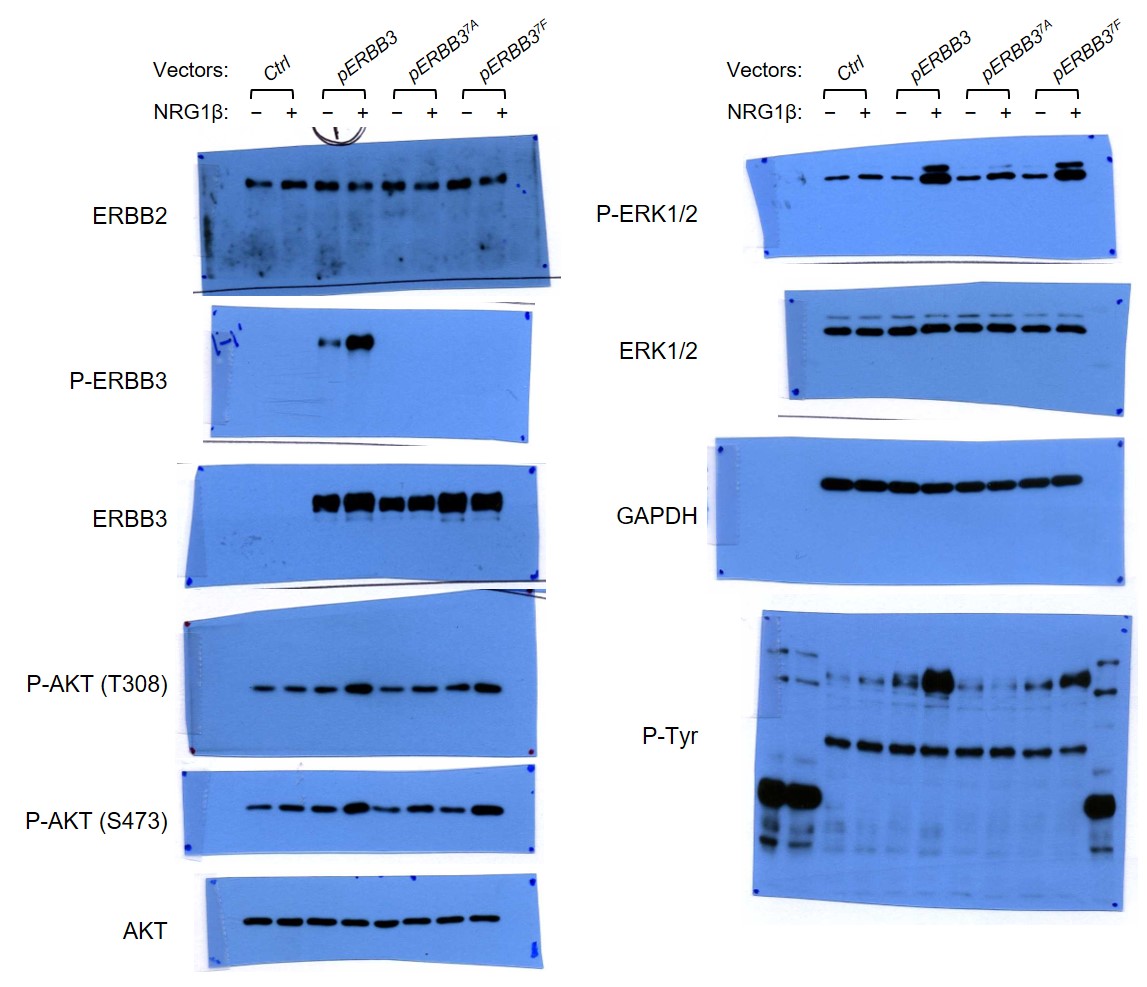


Fig. 6B

| pAKT-308/AKT (n=3) |  |  |  |  |
| --- | --- | --- | --- | --- |
| Ctrl-NRG1β - | 1.00 | 1.00 | 1.00 |  |
| Ctrl-NRG1 + | 1.50 | 1.51 | 1.05 |  |
| pERBB3-NRG1 β - | 2.93 | 2.18 | 1.33 |  |
| pERBB3-NRG1 β + | 7.13 | 3.82 | 2.80 |  |
| pERBB3^7A^- NRG1 β - | 1.11 | 1.15 | 1.22 |  |
| pERBB3^7A^ - NRG1 β + | 2.97 | 1.51 | 1.56 |  |
| pERBB3^7F^ - NRG1 β - | 2.32 | 1.55 | 1.57 |  |
| pERBB3^7F^ - NRG1 β + | 4.50 | 2.77 | 2.88 |  |
| pAKT-473/AKT (n=4) |  |  |  |  |
| Ctrl-NRG1β - | 1.00 | 1.00 | 1.00 | 1.00 |
| Ctrl-NRG1 + | 1.05 | 2.02 | 1.86 | 1.04 |
| pERBB3-NRG1 β - | 2.48 | 2.15 | 2.37 | 1.70 |
| pERBB3-NRG1 β + | 4.72 | 3.96 | 3.44 | 3.17 |
| pERBB3^7A^- NRG1 β - | 1.37 | 1.91 | 1.31 | 0.56 |
| pERBB3^7A^ - NRG1 β + | 2.15 | 3.05 | 2.02 | 1.46 |
| pERBB3^7F^ - NRG1 β - | 1.88 | 1.52 | 1.27 | 0.86 |
| pERBB3^7F^ - NRG1 β + | 2.46 | 4.35 | 4.15 | 3.85 |
| pERK/ERK (n=4) |  |  |  |  |
| Ctrl-NRG1β - | 1.00 | 1.00 | 1.00 | 1.00 |
| Ctrl-NRG1 + | 1.60 | 1.26 | 1.29 | 0.58 |
| pERBB3-NRG1 β - | 2.08 | 0.85 | 0.83 | 0.43 |
| pERBB3-NRG1 β + | 7.82 | 2.96 | 6.00 | 2.74 |
| pERBB3^7A^- NRG1 β - | 2.10 | 0.95 | 0.93 | 0.24 |
| pERBB3^7A^ - NRG1 β + | 3.56 | 1.57 | 1.86 | 0.88 |
| pERBB3^7F^ - NRG1 β - | 1.53 | 1.52 | 0.97 | 0.25 |
| pERBB3^7F^ - NRG1 β + | 5.39 | 4.00 | 5.12 | 3.83 |

Fig. 6C


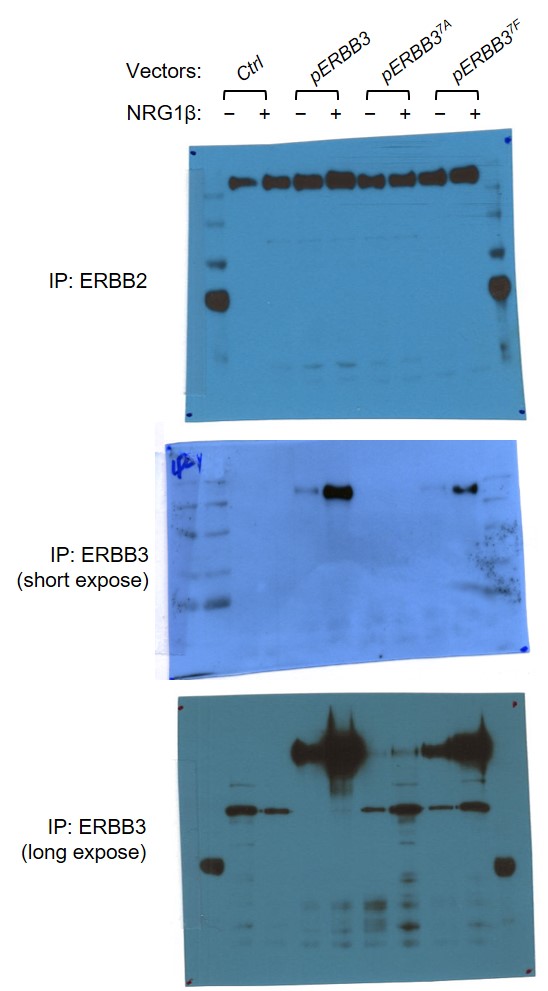

Supplement: S1 File — (DOCX) [file pone.0259426.s001.docx]
