# Supplementary material for: ERBB3-dependent AKT and ERK pathways are essential for atrioventricular cushion development in mouse embryos
Source: PLoS One. 2021 Oct 29;16(10):e0259426. doi: 10.1371/journal.pone.0259426 (PMC8555822; doi:10.1371/journal.pone.0259426)
Supplement: Attachment — Submitted filename: Response to Reviewers_20211005.docx [file pone.0259426.s003.docx]

**Point-by-point Responses**

**We thank the reviewers for their positive and constructive comments and suggestions. In response to their comments, we have accordingly revised and rearranged the manuscript and figures. Please find our point-by-point responses to the reviewers’ constructive criticisms below.**

**Reviewers' comments:**

**Reviewer #2:**

The authors addressed several concerns raised by this reviewer. Specifically, the authors admit their misunderstanding of AV valve morphogenesis and corrected their statements in the manuscript and removed related-figures. The authors also corrected their statements for AV septal morphogenesis. However, there are some other concerns remained to be addressed. The authors also agreed that low cell density in the mutant AV cushions can be explained by the low proliferation ratio of endocardial mesenchymal cells in the mutant AV cushions without having EndMT failure.

The AV explants were dissected out from the embryos at E9.5 and cultured on the collagen gels for 48 hours. The authors show a decrease in mesenchymal cell numbers in the mutant AV cushions cultured on the collagen gels (Fig. 3D). During the 48 hours, newly formed mesenchymal cells proliferate. Proliferation ratio of mesenchymal cells in mutant mice is significantly lower than that of wild type mesenchymal cells (Fig. 4B) at E10.5. Lower cell numbers of mesenchymal cells derived from mutant AV explants and lower cell densities in mutant AV cushion mesenchyme can be simply explained by lower proliferation ratio of mutant AV cushion mesenchymal cells.

In case cell numbers and densities are low but there is no significant difference in cell proliferation ratio between mutant and wild type AV cushion mesenchymal cells, one can claim that there is EndMT defect. In this paper, the authors do not present clear evidence that there are EndMT defects in the mutant AV cushions. The data the authors present in this manuscript show that the mutant mice exhibit defects in AV endocardial cushion development. The abstract should be revised to reflect their findings. Abstract, Page 2, Line 33, Erbb3 knock-in-embryos showed a decrease in the endocardial mesenchymal transformation (EndMT).

The following description is suggested: Erbb3 knock-in-embryos showed a decrease in mesenchymal cell numbers and density in AV cushions.

**Response: We agree with your proposed suggestion and have modified the phrase on p.2, lines 32–34, as follows: “*Erbb3* knock-in embryos exhibited lethality between E12.5 to E13.5, and showed a decrease in mesenchymal cell numbers and density in AV cushions.”**

**Minor issue:**

As the reviewer #3 suggests, the authors have to improve qualities of their figures. According to the figures this reviewer can see through the web, figures appear still very vague and not sharp enough for actual publication.

**Response: Thank you for the helpful feedback. We replaced the original figures with revised figures using PACE to improve their quality.**

**Reviewer #3:**

I appreciate the authors’ efforts to address the changes requested by the reviewers. I have read the new manuscript and there are several issues that still need to be addressed.

1) The sentence, “were caused by overall (Fig. 2C).” on line 223 appears to have been truncated.

**Response: Our apologies for the unintended omission. We have modified the sentence on p.9, lines 221–222, as follows: “Microscopic and histological examination results of E12.5 embryos showed that both *Erbb3^7A/7A^* and *Erbb3^-/-^* embryos have a small right ventricle (Fig. 2B, C).”**

2) Remove the word “Expected” in Figure 2A

**Response: We have removed the word "Expected" from Figure 2A.**

3) If the number of mesenchymal cells is lower in WT and 7a/7a embryos, but the density is the same, then that indicates that the endocardial cushion size in 7A/7A embryos is smaller. Correct?

**Response: Yes, that is correct.**

4) The authors state that, VE-cadherin is a “key player” in EMT. They should state specifically its role in EMT. I assume that in this context, VE-cadherin represses EMT. Correct?

**Response: Thank you for your query. Midgett et al. (Frontiers in Physiology, 2017, doi: 10.3389/fphys.2017.00056.) reported that VE-cadherin connects the junctions between endocardial cells and it is lost when EndMT occurs. Furthermore, VE-cadherin protein expressed in endocardial cells is a recognized endocardial cell marker. Niessen et al. (Circulation Research, 2008, doi: 10.1161/CIRCRESAHA.108.174318.) described a mechanism in which the function of Slug and Snail is similar, both involving the negative regulation of VE-cadherin expression and the initiation of EndMT. In this context, we agree with the reviewer’s position that VE-cadherin is a key player in EndMT and represses EndMT.**

5) For Fig 5A it appears that the authors selected differnet p-AKT and AKT lanes for their analysis of wild-type, heterozygous and null embryos. The GAPDH control lanes were also selected form alternative lanes as well (see Supplemental Material). This is unacceptable.

**Response: We agree with your opinion. We use Western blot data from the same lane in the revised Fig. 5A.**

6) Fig. 5B. Show the E10.5 immunohistochemistry.

**Response: Fig. 5B and C only show the E11.5 immunohistochemistry and there are no images of E10.5 immunohistochemical staining. We performed immunofluorescence staining in both E10.5 and E11.5 embryos to measure the relative intensities of P-AKT and P-ERK proteins, but immunohistochemical staining was performed and imaged only at the representative E11.5 stage.**

7) Fig. 5C. Show the E10.5 immunohistochemistry.

**Response: Although immunofluorescence staining was performed in E10.5 and E11.5 embryos to measure P-AKT and P-ERK protein intensity, immunohistochemical staining was performed and imaged only at the representative E11.5 stage.**

8) I agree that the authors show that, “In both mutants, the right ventricle was smaller than the left ventricle, and the myocardial thickness was reduced.” I however, disagree that these results, “suggest that the heart defects in both the Erbb3-/- and Erbb37A/7A mutants were caused by overall growth retardation.” If that were true, then you would expect an overall reduction in heart size, not in defects in only one ventricle. They may not have a good explanation, but no explanation is better than one that can’t be supported by the data.

**Response: In our results, the right ventricle of both mutants was smaller than the left ventricle and had decreased myocardial thickness. Our results showing defects in cell proliferation and EndMT support this finding. In addition, although not quantified, Figure 2 shows that the heart size from E12.5 to E13.5 is progressively smaller than normal. These findings were the basis why we stated that heart defects in both *Erbb3^-/-^* and *Erbb3^7A/7A^* mutants were caused by the overall growth retardation.**

9) I also note that the change in the right ventricle size compared to the left ventricle is easy to see at E12.5, The authors show no embryo photos at that stage. Instead, the small embryo size is seen at E13.5 when the hearts in section also seem to be overall smaller than wild-type.

**Response: Thank you for the query. In E12.5 embryos, there were no morphological differences between the normal and the two variants. Therefore, the heart sections of E12.5 and E13.5 shown in Figure 2 address the reviewer's concern.**

10) Was the myocardial thickness of the left ventricle affected?

**Response: We found decreased myocardial thickness in both *Erbb3^-/-^* and Erbb3^7A/7A^, but the difference between the left and right myocardial thickness was not significant.**

11) The authors state, “Overall, our results suggest that the YXXA mutation completely blocks the activation of ERBB3-dependent PI3K signaling than does the FXXM mutation of ERBB3.” This is not grammatically correct. Did they mean to say, “

PI3K signaling more so than does the FXXM mutation of ERBB3.”

**Response: The constructive feedback is appreciated. We have modified the phrase on p.14, lines 351–353, as follows: “Overall, our results suggest that the YXXA mutation blocks the activation of ERBB3-dependent PI3K signaling more than the FXXM mutation of ERBB3.”**

12) The authors also state, “Furthermore, our data suggest that ERBB3-dependent PI3K signaling is essential for AV cushion development and valve formation.” The AV cushions develop in ERBB3 null mice, right? Hence, ERRB3 can’t be essential for AV cushion development. In addition, I have seen no evidence of any effect on valve formation in this paper.

**Response: Thank you for raising this important point. We agree and have removed the phrase on p.14, lines 353–354.**
